# *NR1H4* disease: rapidly progressing neonatal intrahepatic cholestasis and early death

**DOI:** 10.1186/s13023-024-03166-1

**Published:** 2024-04-19

**Authors:** Zhong-Die Li, Yu-Chuan Li, Jing-Zhao, Jian-She Wang, Xin-Bao Xie

**Affiliations:** The Center for Pediatric Liver Diseases, Children’s Hospital of Fudan University, No. 399 Wanyuan Road, Minhang District, 201102 Shanghai, China

**Keywords:** *NR1H4*, low-γ-glutamyl transferase (GGT) cholestasis, Liver transplantation

## Abstract

**Background:**

Clinical studies on progressive familial intrahepatic cholestasis (PFIC) type 5 caused by mutations in *NR1H4* are limited.

**Methods:**

New patients with biallelic *NR1H4* variants from our center and all patients from literature were retrospectively analyzed.

**Results:**

Three new patients were identified to be carrying five new variants. Liver phenotypes of our patients manifests as low-γ-glutamyl transferase cholestasis, liver failure and related complications. One patient underwent liver transplantation (LT) and survived, and two other patients died without LT. Nine other patients were collected through literature review. Twelve out of 13 patients showed neonatal jaundice, with the median age of onset being 7 days after birth. Reported clinical manifestations included cholestasis (13/13, 100%), elevated AFP (11/11, 100%), coagulopathy (11/11, 100%), hypoglycemia (9/13, 69%), failure to thrive (8/13, 62%), splenomegaly (7/13, 54%), hyperammonemia (7/13, 54%), and hepatomegaly (6/13, 46%). Six of 13 patients received LT at a median age of 6.2 months, and only one patient died of acute infection at one year after LT. Other 7 patients had no LT and died with a median age of 5 months (range 1.2-8). There were 8 patients with homozygous genotype and 5 patients with compound heterozygous genotype. In total, 13 different variants were detected, and 5 out of 12 single or multiple nucleotides variants were located in exon 5.

**Conclusions:**

We identified three newly-diagnosed patients and five novel mutations. *NR1H4*-related PFIC typically cause progressive disease and early death. LT may be the only lifesaving therapy leading to cure.

**Supplementary Information:**

The online version contains supplementary material available at 10.1186/s13023-024-03166-1.

## Background

Progressive familial intrahepatic cholestasis (PFIC) is an ever-growing group of autosomal recessive liver disorders caused by defects in genes associated with bile secretion, bile salt and lipid transporters and regulators [1, 2]. Disease-causing genes of PFIC were gradually revealed, including *ATP8B1*, *ABCB11*, *ABCB4*, *TJP2*, *NR1H4*, and *MYO5B* (named PFIC1 to 6) [3–6]. *NR1H4* gene (OMIM *603,826), located on 12q23.1, encodes the farnesoid X receptor (FXR), a bile acid (BA)-activated transcription factor, and plays an essential role in BA homeostasis [7]. Biallelic pathogenic variants in *NR1H4* were first identified in low-γ-glutamyl transferase (GGT) cholestasis patients and termed as PFIC5 in 2016 ^4^. So far, only 10 patients with PFIC5 from six unrelated families have been reported, usually presenting as rapidly progressive liver failure, vitamin K independent coagulopathy, high alpha-fetoprotein (AFP) and ultimately required a liver transplant (LT) to save lives [4, 8–10]. To assess phenotypic spectrum and clinical outcomes in *NR1H4*-related PFIC, we studied our patients in detail and reviewed previously reported patients in the literature.

## Methods

### Subjects

Our patients were all Chinese children referred to the Center for Pediatric Liver Disease, Children’s Hospital of Fudan University from February 2016 to March 2023. Genetic testing, either whole-exome sequencing or liver panel sequencing, was performed in patients after excluding other causes of liver diseases (including infection, drug exposure, autoimmune hepatitis, and biliary atresia) [11]. Cytomegalovirus infection was not excluded due to its high prevalence in Chinese infants. When other known inherited liver disorders were excluded, patients with two or biallelic *NR1H4* pathogenic/likely pathogenic/uncertain significance (P/LP/US) variants were collected. P/LP/US variants were classified according to the American College of Medical Genetics (ACMG)/Association for Molecular Pathology (AMP) criteria [12].

### Genetic analyses and *in silico* assessment of *NR1H4* variants

We confirmed these variants by Sanger sequencing and confirmed parental origins when available. Assessment of variant pathogenicity were performed with seven *in silico* tools including MutationTaster (http://www.mutationtaster.org/), Sorting Intolerant From Tolerant (SIFT, http://sift.jcvi.org), Rare Exome Variant Ensemble Learner (REVEL, https://labworm.com/tool/revel), MutPred (http://mutpred.mutdb.org/), and Protein Variation Effect Analyzer (PROVEAN, http://provean.jcvi.org/index.php). Two programs, SpliceAI (https://spliceailookup.broadinstitute.org/) and varSEAK (https://varseak.bio/), were used to evaluate the effect of variants on mRNA splicing. Default settings were used for all *in silico* tools.

### Literature review

A comprehensive literature review of the current literature was performed on March 2023 by searching PubMed (https://pubmed.ncbi.nlm.nih.gov/), CNKI (https://www.cnki.net/), and Wan fang (https://www.wanfangdata.com.cn/) databases using the keywords “*NR1H4* variants, progressive familial intrahepatic cholestasis 5, and PFIC5”.

## Results

### Identification of biallelic *NR1H4* variants in 3 new patients and *in silico* assessment

We identified 5 unique *NR1H4* variants from 3 unrelated Chinese patients with low GGT intrahepatic cholestasis from our cohort. Among these, there were 3 missense variants, one nonsense variant, and one canonical splicing variant. All variants have not been previously reported in medical literatures (See Table 1). Three out of 5 variants were absent in gnomAD (c.505T > A/p. (Cys169Ser), c.1235T > C/p. (Leu412Pro), and c.1066 + 1G > A/p.?). The other two were present in gnomAD, with a population frequency of 0/1/251,458 (number of homozygotes/allele count/allele number) for the c.688 C > T/p. (Arg230Ter) variant and 0/1/31,410 (number of homozygotes/allele count/allele number) for the c.527G > A/p. (Arg176Gln) variant. MutationTaster predicted that the nonsense variant (c.688 C > T/p. (Arg230Ter)) may lead to nonsense-mediated mRNA decay (NMD). SpliceAI and varSEAK predicted that the canonical splicing variant (c.1066 + 1G > A/p.?) lead to loss of donor splice site, most likely leading to protein truncation. Three missense variants were predicted to be pathogenic by five pathogenicity prediction tools, and have no effect on pre-mRNA splicing. According to the ACMG/AMP criteria, all of the variants were classified as P/LP/US (Table 1).

**Table 1:** *NR1H4* variants and the results of in silico pathogenicity prediction

Patients	cDNA change (NM_005123.4)	Protein change (NP_005114.1)	Parental origin	gnomAD	gnomAD EAS	MuT	SIFT	REVEL	MutPred	PROVEAN	SpliceAI	varSEAK	ACMG grade
P1	c.688 C > T	p.Arg230Ter (het)	maternal	0 / 1 / 251,458	0 / 0 / 18,394	NMD	/	/	/	/	N	N	P: PVS1 + PM2_S + PP4
	c.505T > A	p.Cys169Ser (het)	paternal	-	-	D	D	D	D	D	N	N	LP: PP3 + PM2_S + PP4 + PM3
P2	c.1235T > C	p.Leu412Pro (hom)	n.a.	-	-	D	D	D	D	D	N	N	LP: PP3 + PM2_S + PP4
P3	c.1066 + 1G > A	/	maternal	-	-	D	/	/	/	/	D	D	P: PVS1 + PM2_S + PP4
	c.527G > A	p.Arg176Gln	paternal	0 / 1 / 31,410	0 / 0 / 1560	D	D	D	D	D	N	N	LP: PP3 + PM2_S + PP4 + PM3

gnomAD and gnomAD EAS: frequencies of corresponding mutations respectively in all populations and in East Asian populations of gnomAD (http://gnomad-old.broadinstitute.org/); MuT: MutationTaster (http://www.mutationtaster.org); SIFT: Sorting Intolerant From Tolerant (http://provean.jcvi.org/index.php); REVEL: Rare Exome Variant Ensemble Learner (https://sites.google.com/site/revelgenomics/); MutPred: http://mutpred.mutdb.org/; PROVEAN: Protein Variation Effect Analyzer (http://provean.jcvi.org/index.php; SpliceAI: https://spliceailookup.broadinstitute.org/; varSEAK, https://varseak.bio/index.php?hascredit=false; ACMG, the American College of Medical Genetics and Genomics (http://acmg.cbgc.org.cn); P1-3, patient 1–3; NMD, nonsense-mediated mRNA decay; /, not applicable; N, no effect; P, pathogenic; PVS1, null variants includes nonsense, frameshift, canonical ± 1 or 2 splice sites, initiation codon, single exon or multi-exon deletion variants; PM2_S, variants are absent in gnomAD; PP4, clinical features matches the known clinical phenotype of *NR1H4* disease; -, variants were absent in gnomAD and gnomAD EAS; D, disease-causing; LP, likely pathogenic; PM3, variants occur in trans; n.a., not available

### Clinical features and outcome of 3 new patients with *NR1H4*-related cholestasis

All 3 male patients born at full-term with normal weight to non-consanguineous parents following uneventful pregnancies. The first child in family II died of unexplained liver disorders at the age of 7 months, whereas the other families did not have positive family history. All of them presented with jaundice during the first few days after birth. Normal growth and development were observed in patient 1(P1), and failure-to-thrive occurred in patients 2 (P2) and 3 (P3). Our patients have similar clinical features resembling previously reported patients, including low-GGT cholestasis, rapidly progressive liver failure/decompensated cirrhosis, vitamin K independent coagulopathy, and markedly elevated AFP levels (Table 2). Initial, pre-transplantation, or pre-death laboratory testing results were shown in Table 3. In addition, our patients had recurrent severe pneumonia, splenomegaly, and elevated urinary microalbumin. Two patients (P1 and P3) had hepatomegaly and hydrocele.

**Table 2:** Clinical features and outcomes of our 3 patients with NR1H4-related cholestasis

ID	Patient 1 (Family I)	Patient 2 (Family II)	Patient 3 (Family III)
Sex	male	male	male
Consanguinity	no	no	no
Family history	no	yes	no
Gestational week	full term	full term	full term
Birth weight (kg)	3.50	4.20	3.85
Age at onset	5.0 days	3.0 days	2.0 days
Age at the first visit	3.0 months	3.0 months	2.5 months
Height	P25th	< P1th	< P1th
Weight	P50th	< P1th	< P1th
Age at last visit	1.6 years	3.0 months	3.0 months
Initial symptom	jaundice	jaundice	jaundice
Hepatic phenotype	low-γ-glutamyl transferase cholestasis, hepatomegaly, and decompensated cirrhosis	low-γ-glutamyl transferase cholestasis and acute liver failure	low-γ-glutamyl transferase cholestasis, acute liver failure, and hepatomegaly
Age at liver biopsy	6.0 months (after liver transplantation)	/	3.0 months
Biopsy findings	swelling of hepatocytes, lymphocytic infiltration, and mild fibrous tissue proliferation	/	hepatocyte ballooning, multi-nucleated giant cells, cholestasis, fibrous tissue proliferation, infiltration of inflammatory cells, bile duct proliferation, hepatocellular necrosis, and decreased BESP expression
Extra-hepatic phenotype	before LT: severe pneumonia, splenomegaly, hydrocele, massive ascites, elevated urinary microalbumin after LT: EBV infection, bowel obstruction, and right-sided diaphragmatic hernia	severe pneumonia, splenomegaly, and elevated urinary microalbumin	severe pneumonia, cholecystitis, splenomegaly, elevated urinary microalbumin, hyperammonemia, and hydrocele
Liver transplantation (LT)	yes	no	no
Age at LT	4.0 months	/	/
Outcomes	normal liver function at one year’s post-transplant	died of infection at 3 months of age	died of infection at 3 months of age

**Table 3:** Initial and pre-transplantation/pre-death laboratory testing of our 3 patients with NR1H4-related cholestasis

ID	Patient 1		Patient 2		Patient3	
Time to evaluation	Initial visit	Before LT	Initial visit	Before death	Initial visit	Before death
Serum biochemistry (reference range)						
Total bilirubin (5.1–17.1 umol/l)	180	189	348	273	304	278
Direct bilirubin (0–6 umol/l)	118	126	236	209	235	233
Alanine aminotransferase (0–40 IU/L)	117	95	270	227	259	260
Aspartate aminotransferase (0–40 IU/L)	211	131	473	363	445	427
γ-glutamyl transferase (7-50IU/L)	26	33	45	40	43	40
Total bile acid (0-10umol/l)	21	81	243	132	235	296
Albumin (35–55 g/l)	33	43	43	31	35	34
Triglyceride (0.56–1.7 mmol/l)	0.24	n.a.	0.75	0.77	0.70	0.74
Total cholesterol (3.1-5.2mmol/l)	2.42	n.a.	3.65	3.95	4.35	3.10
25-hydroxyvitamin D3 (0–20 ng/ml)	6.07	n.a.	7.08	n.a.	12.14	n.a.
Alpha-fetoprotein (0-3.7 ng/ml)	> 121,000	n.a.	> 121,000	n.a.	>121,000	n.a.
Ammonia (10–47 umol/l)	73	63	115	93	93	131
Blood coagulation (reference range)						
Platelets(×10^9/L)	413	342	170	180	335	399
Prothrombin time (12.0–14.8 s)	21.1	22.2	23.8	24.4	21.5	25.2
International normalized ratio (0.8–1.2 s)	1.78	1.91	2.10	2.20	1.85	2.25

LT, liver transplantation; n.a., not available

The patient P1 suffered massive ascites from decompensated cirrhosis prior to liver transplantation (LT). He had a positive plasma Epstein-Barr virus DNA from 2 months post-LT until the last visit. He also underwent a single surgery due to small bowel obstruction and right-sided diaphragmatic hernia one year after LT. The patient P3 presented with hyperammonemia due to acute liver failure (ALF) and his magnetic resonance of the brain showed minor abnormalities such as widened extracerebral space and cavum septum pellucidum.

Only one patient (P1) was treated by LT at the age of 4 months, and the other two patients died of infection at 3 months of age. Up to the latest assessment, P1 had normal liver function at one-year post-transplant.

### Clinical and genetic characterization of 13 *NR1H4*-related PFIC patients

A total of 13 patients from 10 unrelated families were collected, with 3 new and 10 reported patients (Supplementary Table 1) [4, 8–10]. Eight were males, 4 were females, and one patient’s gender was unknown. All patients were delivered at full term without maternal or fetal complications. The age of onset ranged from the neonatal period to 17 months, with the median age of onset being 7 days after birth. Twelve out of 13 patients showed neonatal jaundice in the early neonatal period (7 patients, 0–6 days) and late neonatal period (5 patients, 7–28 days), respectively. Only one patient was admitted to hospital due to jaundice and abdominal distention at the age of 17 months. Reported clinical manifestations include: cholestasis (13/13, 100%), persistently elevated AFP (11/11, 100%), coagulopathy (11/11, 100%), hypoglycemia (9/13, 69%), splenomegaly (7/13, 54%), hyperammonemia (7/13, 54%), failure to thrive (8/13, 62%), and hepatomegaly (6/13, 46%). Six of 13 patients received LT at a median age of 6.2 months (range 2–20). Only one patient died of acute infection at one year after LT. Five out of 6 patients with LT are still alive, with a median age of 6 years (range 1.3–10). Other 7 patients without LT died at a median age of 5 months (range 1.2-8). The causes of death included ALF, multiple organ dysfunction syndrome (MODS), sepsis, and others (Supplementary Table 1).

**Fig. 1:**
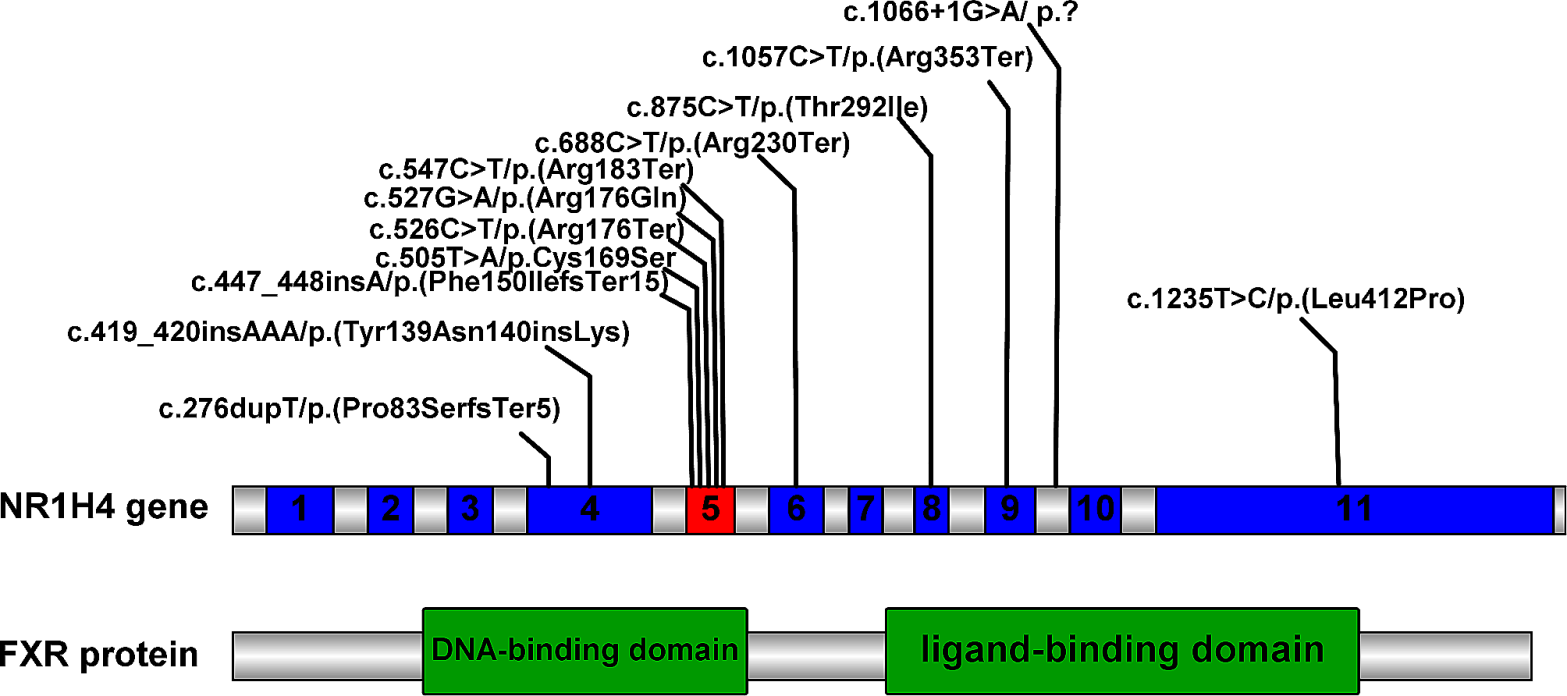
Schematic presentation of the farnesoid X receptor (FXR) protein and locations of 12 unique single or multiple nucleotides variants identified in our cohort and literature. The exon 5 was depicted in red, and other exons of the *NR1H4* gene are depicted in blue; FXR protein domains are shown in green

Eight patients had homozygous genotype and 5 patients had compound heterozygous genotype. In total, 13 different variants were detected, including 4 nonsense variants, 4 missense variants, 2 frameshift variants, one splice site variant, one in-frame insertion variant, and one large DNA fragment deletion variant. Overall, there were five variants (41.6%) in exon 5, two variants (16.7%) in exon 4, one variant (8.3%) in exon 6, one variant (8.3%) in exon 8, one variant (8.3%) in exon 9, one variant (8.3%) in intron 9 and one variant (8.3%) in exon 11 (Figure 1).

### Liver pathological characteristics in *NR1H4*-related PFIC patients

Liver pathology was documented in 7 of 13 *NR1H4*-related PFIC [8, 9]. Characteristic pathological features included cholestasis, steatosis, micro-nodular cirrhosis, hepatocellular ballooning, fibrous tissue proliferation, fibrosis, inflammatory cell infiltration, and proliferation of bile ducts. Immunohistochemical stainings of both bile salt export pump (BESP) and FXR proteins were absent, and the multi-drug resistance protein 3 (MDR3) expression was decreased or normal in all 7 patients.

## Discussion

It was first discovered 7 years ago that the *NR1H4* was responsible for PFIC [4]. Previous studies showed that the *NR1H4*-associated PFIC had early-onset and rapid disease progression with high mortality [4, 8]. As only few patients have been reported, current understanding of PFIC5 caused by *NR1H4* defect is limited. Therefore, we performed a retrospective analysis to obtain a better understanding of clinical phenotype and outcomes of PFIC5 caused by *NR1H4* in our center and literature.

The FXR, encoded by *NR1H4* gene, as the master regulator of BA homeostasis, regulates BA homeostasis, biliary BA secretion, and intestinal re-absorption [13–15]. Compared with other PFIC patients, the PFIC5 patients caused by *NR1H4* defect had significantly worse prognosis due to more rapid progression [8, 16, 17]. All patients without LT died and survival with native liver has not been observed. Three new patients in our cohort exhibited similar clinical characteristics as published case [7, 8]. The liver phenotypes of all reported patients were extremely similar and presented as low GGT neonatal cholestasis with rapid progression to ALF (with/without related complications such as hypoglycemia, hyperammonemia, coagulopathy, hepatosplenomegaly, hydrothorax, and ascites) [8, 9].

While *NR1H4* gene is predominantly expressed in liver and intestine, it also presents in kidney, spleen, heart, gallbladder, pancreas, adrenal glands, bone marrow, and other tissues [18–21]. We further summarized the extra-hepatic phenotypes of 13 patients. Of those, the failure to thrive was the most common finding. Other extrahepatic manifestations were also described, such as atrial septal defect, butterfly vertebra, decreased bone mineral density, intestinal obstruction, diaphragmatic hernia, inguinal hernia, and iris coloboma. Although *NR1H4* is highly expressed in the intestine, recurrent or severe diarrhea has not been observed in patients with *NR1H4*-related disorder [8–10].

Notably, pharmacological therapy is typically not effective for *NR1H4* disease [8]. Those patients without LT died in early infancy, and the common causes of death included ALF, MODS, and severe infection [8]. Therefore, LT may be the only curative option. Fortunately, 5 out of 6 patients are still alive after LT without serious postoperative complications, and with good clinical outcome during the follow-up. Only one patient died of acute infections one month after the transplant [9], this suggests that minimizing the risk of infection is the key to reduce morbidity and mortality associated with LT for PFIC5 patients [22, 23].

All enrolled patients had poor prognosis with native liver. So, we were not able to analyze the relationship between genotypes and phenotype. However, we observed that 41.6% of all reported variants are located on the exon 5 of *NR1H4* gene. Exon5 encodes a highly conserved DNA binding domain of FXR by binding to specific DNA sequences called hormone response elements, thereby possibly regulating other gene expression [24, 25]. More cases and further studies are needed to confirm whether exon 5 is a susceptible or hotspot region for *NR1H4* gene mutation.

## Conclusions

*NR1H4*-related PFIC is characterized by severe neonatal cholestasis, rapid progression to liver failure, and early death. LT might be the only lifesaving therapy that can lead to cure. At present, no severe complications of LT related to *NR1H4* gene were observed, but long-term outcome of LT still needs to be validated in more patients.

### Electronic supplementary material

Below is the link to the electronic supplementary material.

Supplementary Material 1

## Data Availability

All data generated or analyzed during this study are included in this published article and its supplementary information files.

## Abbreviations

AMP: Association for Molecular Pathology
ACMG: American College of Medical Genetics
ALF: Acute liver failure
AFP: High alpha-fetoprotein
BA: Bile acid
BESP: Bile salt export pump
FXR: Farnesoid X receptor
GGT: Glutamyl transferase
LP: Likely pathogenic
LT: Liver transplantation
MDR3: Multi-drug resistance protein 3
NMD: Nonsense-mediated mRNA decay
P: Pathogenic
PFIC: Progressive familial intrahepatic cholestasis
US: Uncertain significant
