# Supplementary material for: NR1H4 disease: rapidly progressing neonatal intrahepatic cholestasis and early death
Source: Orphanet J Rare Dis. 2024 Apr 19;19:171. doi: 10.1186/s13023-024-03166-1 (PMC11027519; doi:10.1186/s13023-024-03166-1)
Supplement: Supplementary file 1 — Supplementary Material 1 [file 13023_2024_3166_MOESM1_ESM.docx]

Supplementary table 1. Phenotype and genotype of 13 patients with *NR1H4*-related cholestasis

| Patients | 1 | 2 | 3 | 4 | 5 | 6 | 7 | 8 | 9 | 10 | 11 | 12 | 13 |
| --- | --- | --- | --- | --- | --- | --- | --- | --- | --- | --- | --- | --- | --- |
| Families | I | I | II | II | III | IV | V | V | VI | VII | VIII | IV | X |
| Sex | female | male | female | male | female | male | female | male | male | n.a. | male | male | male |
| Consanguinity | + | + | - | - | - | + | + | - | - | + | - | - | - |
| Pregnancy | - | - | - | - | - | - | - | - | - | - | - | - | - |
| Gestational week | full term | full term | full term | full term | full term | full term | full term | full term | full term | Full term | full term | full term | full term |
| Age at onset | 2 weeks | 2 weeks | 6 weeks | after birth | after birth | 17 months | 3 weeks | 1 week | 2 days | 3 days | 5 days | 3 days | 2 days |
| Initial symptom | jaundice | jaundice | jaundice | jaundice | jaundice | jaundice, abdominal distension, FTT | jaundice, FTT, respiratory distress related with hydrothorax | jaundice, FTT, respiratory distress related with hydrothorax | jaundice | jaundice | jaundice | jaundice | jaundice |
| Cholestasis | + | + | + | + | + | + | + | + | + | + | + | + | + |
| Hepatomegaly | - | - | - | - | - | + | - | + | + | - | + | + | + |
| Splenomegaly | - | - | - | - | + | + | - | + | + (splenectomy) | - | + | + | + |
| Hypoglycemia | + | + | + | - | - | + | + | - | + | - | + | + | + |
| Elevated AFP | + | + | + | + | + | + | n.a. | + | + | n.a. | + | + | + |
| Decreased BMD | n.a. | n.a. | n.a. | n.a. | n.a. | + | n.a. | n.a. | - | - | - | - | - |
| Hyperammonemia | + | + | + | - | - | - | + | - | - | - | + | + | + |
| Coagulopathy | + | + | + | + | + | n.a. | n.a. | n.a. | + | + | + | + | + |
| Failure to thrive | + | + | - | - | - | - | + | + | + | + | - | + | + |
| Others | - | - | - | IH, hydrothorax  and ascites | - | - | hydrothorax | hydrothorax | ASD, butterfly vertebra, inguinal hernia, iridial coloboma and hypercholesterolemia | CMV infection, ascites，dysphagia, dystrophy, acidosis, proteinuria, intermittent diarrhoea, delayed speech development | EBV infection,severe pneumonia,hydrocele, massive ascites, EUM, bowel obstruction, right-sided diaphragmatic hernia | severe pneumonia, EUM | cholecystitis, EUM, hydrocele |
| LT | + | + | - | - | - | + | - | - | + | + | + | - | - |
| Age at LT | 2.0 months | 4.4 months | - | - | - | 20 months | - | - | 8 months | 8 months | 4 months | - | - |
| Age at the last visit | 10 years | 15 months | 8 months | 5 weeks | 5 months | 7.6 years | 8 months | 7 months | 1.6 years | 6 years | 1.6 years | 3 months | 3 months |
| Outcomes | survival | survival | died of LF at 8 months of age | died of aortic thrombus and IH at 5 weeks of age | died of sepsis at 5 months of age | survival | died of MODS at 8 months of age | died of LF at 7 months of age | died of acute infection at acute infection at one year after LT | survival | survival | died of infection at 3 months of age | died of infection at 3 months of age |
| Genotype | hom | hom | CH | CH | CH | hom | hom | hom | hom | hom | CH | hom | CH |
| Variant 1 | c.526C>T/  p.(Arg176Ter) | c.526C>T/  p.(Arg176Ter) | c.419_420insAAA/  p.(Tyr139_Asn140insLys) | c.419_420insAAA/ p.(Tyr139Asn140insLys) | c.447_448insA/p.(Phe150IlefsTer15) | c.526C>T/ p.(Arg176Ter) | c.276dup p.(Pro83SerfsTer5) | c.276dup/ p.(Pro83SerfsTer5) | c.547C>T/  p.(Arg183Ter) | c.875C>T/  p.(Thr292Ile) | c.688C>T/  p.(Arg230Ter) | c.1235T>C/  p.(Leu412Pro) | c.1066+1G>A/  p.? |
| Variant 2 | c.526C>T/  p.(Arg176Ter) | c.526C>T  p.(Arg176Ter) | 31.7 Kb deletion | 31.7 Kb deletion | c.1057C>T/  p.(Arg353Ter) | c.526C>T/ p.(Arg176Ter) | c.276dup p.(Pro83SerfsTer5) | c.276dup/ p.(Pro83SerfsTer5) | c.547C>T/  p.(Arg183Ter) | c.875C>T/  p.(Thr292Ile) | c.505T>A/  p.(Cys169Ser) | c.1235T>C/  p.(Leu412Pro) | c.527G>A/ p.(Arg176Gln) |
| Reference | [4] | [4] | [4] | [4] | [10] | [8] | [8] | [8] | [9] | [17] | Our study | Our study | Our study |

+, present; -, not present; n.a., not available; AFP, alpha-fetoprotein, BMD, bone mineral density, FTT, failure to thrive; IH, intraventricular hemorrhage; ASD, atrial septal defect; CMV, Cytomegalovirus; EBV, Epstein-Barr virus; EUM, elevated urinary microalbumin; LT, liver transplantation; LF, liver failure; MODS, multiple organ dysfunction syndrome; hom, homozygosity; CH, compound heterozygosity.
